# The Effect of Lactic Acid Bacteria Fermentation on the Anti-Diabetic Activity of Pumpkin Puree

**DOI:** 10.3390/foods15111882

**Published:** 2026-05-26

**Authors:** Aqsa Qayyum, Shahid Ahmed Junejo, Zuoting Xu, Muhammad Zubair Hassan, Bingjie Liu, Zhong Chen

**Affiliations:** 1 School of Food Science and Engineering, South China University of Technology, Wushan Road 381, Guangzhou 510640, China; aqsaabdulqayyum4@gmail.com (A.Q.); shahidfst42@gmail.com (S.A.J.); fetunmux@mail.edu.cn (Z.X.); zh4917614@gmail.com (M.Z.H.); 2 Dongguan Shilong Jinwei Beverage & Food Co., Ltd., Dongguan 523320, China; lbjie001@163.com

**Keywords:** pumpkin fermentation, lactic acid bacteria, diabetes mellitus, antioxidants, glucose uptake, bioactive compounds

## Abstract

The purpose of this study was to evaluate the effects of multi-strain lactic acid bacteria (LAB) fermentation on the functional and antidiabetic properties of pumpkin (*Cucurbita moschata*) puree using integrated physicochemical, biochemical, and cellular analyses. Fermentation induced significant (*p* < 0.05) physiochemical changes, including a decrease in pH from 6.2 to 6.5 to 3.5–3.6, increased titratable acidity, and higher viable cell counts, indicating active microbial fermentation. Levels of reducing and soluble sugars (glucose, fructose, sucrose, and maltose) decreased significantly due to microbial utilization during fermentation. Fermented pumpkin puree exhibited markedly enhanced antioxidant activity, with DPPH radical scavenging activity increasing from 45% in the control to 83.2%, while ABTS radical scavenging activity increased from 33% to 42%. *In vitro* enzyme inhibition assays demonstrated enhanced antidiabetic potential, with α-amylase inhibition increasing from 7% to 60% and α-glucosidase inhibition from 10% to 70%. Moreover, glucose uptake in insulin-resistant L6 myotubes was significantly enhanced, indicating improved cellular glucose utilization. HPLC analysis revealed significant enrichment of phenolic compounds, particularly trans-ferulic acid (3894 µg/g), gallic acid (1996 µg/g), and caffeic acid (1894 µg/g), suggesting microbial-mediated release and biotransformation of bound phenolics during fermentation. Correlation analysis showed strong positive relationships among phenolic content, antioxidant activity, and enzyme inhibition. Among the tested LAB strains, *Lactobacillus plantarum* and *Lactobacillus paracasei* competitively exhibited the highest functional and anti-diabetic properties. Overall, LAB fermentation effectively enhanced the functional and antidiabetic properties of pumpkin puree.

## 1. Introduction

Diabetes mellitus (DM) is a core global health challenge characterized by chronic hyperglycemia and correlated metabolic disturbances. The International Diabetes Federation estimates that approximately 537 million adults are currently alive with diabetes worldwide, and this number is expected to reach 784 million by 2045 [1]. Persistent hyperglycemia contributes to the promotion of severe problems, including retinopathy, nephropathy, neuropathy, and cardiovascular diseases. Type II diabetes mellitus (T2DM), which accounts for more than 90% of diabetes cases, is primarily characterized by insulin resistance and progressive pancreatic β-cell dysfunction, leading to impaired glucose homeostasis and elevated cardiovascular risk [2]. In disparity, type 1 diabetes mellitus results from autoimmune obliteration of pancreatic β-cells and typically develops during childhood or adolescence [3,4]. Current therapeutic plans mainly rely on glucose-lowering drugs and insulin therapy. Although these treatments are effective in controlling blood glucose levels, they are often associated with limitations such as gastrointestinal discomfort, hypoglycemia, weight gain, and long-term economic burden. Moreover, pharmacological approaches primarily focus on glycemic control rather than addressing unique metabolic dysfunction. Accordingly, increasing attention has been paid to dietary strategies and functional foods that may improve glycemic regulation. Plant-derived foods rich in phenolic compounds, flavonoids, and other bioactive constituents have shown potential antihyperglycemic and antioxidant properties, suggesting their possible role as complementary dietary approaches for diabetes management [5,6].

Pumpkin (*Cucurbita moschata*), a member of the family Cucurbitaceae, is widely cultivated and used worldwide. Pumpkin pulp contains diverse bioactive components, including polysaccharides, phenolic acids, flavonoids, vitamins, and minerals, which contribute to its reported antioxidant, anti-inflammatory, and hypoglycemic activities [7,8]. Also, pumpkin is a low-energy food (approximately 30 kcal/100 g), making it a promising dietary ingredient for individuals with metabolic disorders [9,10]. However, most previous studies have focused on unfermented pumpkin extracts or isolated compounds, while limited attention has been given to processing strategies that improve the bioavailability and functionality of pumpkin bioactive compounds [11].

Among such strategies, fermentation has occurred as an effective approach to progress the nutritional value and functional properties of plant-based foods [12]. During fermentation, microorganisms metabolize carbohydrates and other macromolecules while producing organic acids and bioactive metabolites [13]. Lactic acid bacteria (LAB) are extensively used in vegetable and fruit fermentation due to their ability to hydrolyze complex plant components and release bound phenolic compounds through enzymatic activities such as β-glucosidase and esterase [14]. These processes can increase phenolic availability, enhance antioxidant activity, and generate metabolites capable of inhibiting carbohydrate-hydrolyzing enzymes such as α-glucosidase and α-amylase [12]. Importantly, the functional outcomes of fermentation are highly strain dependent because different LAB strains exhibit distinct metabolic capabilities, carbohydrate utilization pathways, and enzyme production profiles [15]. Although LAB fermentation has been extensively studied to improve the functional properties of cereals, legumes, and vegetables, studies on LAB-fermented pumpkin remain limited. Existing research has primarily focused on physicochemical characteristics and fermentation optimization, often using single microbial strains without systematic comparison [11,16]. Moreover, few studies have investigated the relationship between fermentation-induced compositional alterations and biological antidiabetic activities, particularly at the cellular level [17].

Subsequently, this study aimed to evaluate the antidiabetic potential of pumpkin puree fermented with six different lactic acid bacteria (LAB) strains. Fermentation dynamics were monitored by measuring pH, titratable acidity, reducing sugars, and microbial growth. In addition, the effects of fermentation on phenolic composition, antioxidant capacity (DPPH and ABTS assays), and the inhibition of carbohydrate-digesting enzymes (α-glucosidase and α-amylase) were investigated. Furthermore, the biological activity of fermented pumpkin extracts was assessed using an insulin-resistant L6 myotube model to evaluate insulin-stimulated glucose uptake. This study provides a comparative evaluation of multi-strain LAB fermentation of pumpkin and offers insights into its potential application as a functional food for glycemic management.

## 2. Materials and Methods

### 2.1. Materials and Chemicals

In Guangzhou, China, fresh pumpkin (Cucurbita moschata) fruits were purchased from a local marketplace. Six lactic acid bacteria (LAB) strains—*Lactiplantibacillus plantarum* (LP), *Lactobacillus delbrueckii* subsp. *bulgaricus* (LB), *Lacticaseibacillus paracasei* (LPC), *Lacticaseibacillus rhamnosus* GG (LGG), *Lacticaseibacillus casei* (LC), and *Lactobacillus acidophilus* (LA) were obtained from the School of Food Science and Engineering, South China University of Technology, Guangzhou, China [18]. These strains were selected because they are commonly used in food fermentation and have reported potential for acid production, phenolic transformation, antioxidant enhancement, and improvement of functional properties in plant-based matrices. Lyophilized bacterial cultures were stored at –20 °C until activation. De Man–Rogosa–Sharpe (MRS) agar and broth used for LAB cultivation were purchased from Guangdong Huankai Microbial Sci. & Tech. Co., Ltd. (Guangzhou, China). The chemicals 3,5-dinitrosalicylic acid (DNS), 2,2-diphenyl-1-picrylhydrazyl (DPPH), and 2,2′-azino-bis (3-ethylbenzothiazoline-6-sulfonic acid) (ABTS), as well as α-glucosidase and α-amylase enzymes, were purchased from Sigma-Aldrich (St. Louis, MO, USA). In antioxidant assays, ascorbic acid was used as the reference standard. The L6 rat skeletal muscle myoblast cell line (ATCC CRL-1458) used in this study was obtained from the College of Food Science and Engineering, South China University of Technology (Guangzhou, China) and cultured according to the supplier’s recommended protocols.

### 2.2. Preparation of Pumpkin Puree and Inoculated Fermentation

Fresh pumpkins were washed with distilled water, peeled, and deseeded. The edible flesh was cut into small pieces and homogenized using a laboratory mixer (Midea MJ-WBL2501B, Guangzhou, China) to obtain a uniform slurry. To inactivate endogenous enzymes and reduce background microbial load, the slurry was thermally treated at 95–100 °C for 15 min and subsequently cooled to ambient temperature under aseptic conditions. Before fermentation, LAB strains were activated in MRS broth at 37 °C for 12–24 h. The cultures were harvested by centrifugation at 5000× *g* for 10 min and resuspended in sterile saline solution (0.85% NaCl) to obtain an inoculum with a final cell density of approximately 10^7^–10^8^ CFU/mL. Each LAB strain was inoculated separately into 400 mL of pumpkin slurry at 2% (*v*/*v*) and gently mixed to ensure uniform distribution. The inoculum level was selected to provide sufficient initial viable cell counts for reproducible fermentation and comparison among strains. The initial pH of the pumpkin slurry was 6.0 ± 0.1, as measured using a calibrated pH meter, and no adjustment was performed before fermentation to allow evaluation of the natural acidification behavior. After inoculation, 50 mL aliquots were transferred into sterile 100 mL Erlenmeyer flasks. The flasks were sealed with sterile breathable closures and incubated statically at 37 °C for 48 h. Static incubation was employed due to the semi-solid nature of the pumpkin matrix and the predominantly low-oxygen conditions required for LAB fermentation. The incubation temperature (37 °C) was selected to support optimal growth and metabolic activity of the LAB strains. Samples were collected at 0, 6, 12, 24, 30, 36, and 48 h to monitor early, intermediate, and late fermentation stages, including microbial adaptation, acidification, sugar utilization, and bioactive compound transformation. At each sampling point, fermentation was terminated by immediate cooling in an ice bath, and samples were subsequently stored at −20 °C until further analysis. Heat-treated, non-inoculated pumpkin slurry incubated under identical conditions served as the control.

### 2.3. Determination of Viable LAB Counts

Viable LAB counts during fermentation of pumpkin puree were determined using the standard plate count method according to a previously described method [16]. Samples were collected immediately after inoculation (0 h) and at 6, 12, 24, 30, 36, and 48 h of fermentation. The 0 h sample was used to confirm the initial viable count after inoculation. Before enumeration, each sample was homogenized thoroughly. Briefly, 1 g of pumpkin puree was mixed with 9 mL of sterile physiological saline solution (0.85% NaCl) to prepare a 10^−1^ dilution, followed by serial tenfold dilutions, to obtain an inoculum concentration of approximately 10^7^–10^8^ CFU/mL. Aliquots of 100 µL from appropriate dilutions were spread onto MRS agar plates. The plates were incubated anaerobically at 37 °C for 48 h using anaerobic jars with gas-generating sachets. Plates containing 30–300 colonies were selected for enumeration. Colonies were counted using a ProtoCOL 3 automated colony counter (Synbiosis, Frederick, MD, USA), and viable LAB counts were expressed as log CFU/g fresh pumpkin puree.

### 2.4. Physicochemical Analyses

#### 2.4.1. pH Measurement

The pH of fermented pumpkin puree samples was measured using a digital pH meter (S-210, Mettler Toledo, Greifensee, Switzerland) equipped with automatic temperature compensation [16]. Before sample measurement, the pH meter was calibrated at 25 °C using standard buffer solutions of pH 4.01 and 7.00. For measurement, 10 g of each pumpkin puree sample was homogenized with 10 mL of distilled water at a ratio of 1:1 (*w*/*v*). After thorough mixing, the pH of the homogenate was measured using the calibrated pH meter. All measurements were performed in triplicate, and the results were expressed as mean ± standard deviation.

#### 2.4.2. Total Titratable Acidity

Total titratable acidity (TA) of fermented pumpkin puree samples was determined by potentiometric titration according to standard methods for fermented food analysis [19] with minor alterations. Briefly, 10 g of each pumpkin puree sample was mixed with distilled water and diluted to a final volume of 50 mL. The homogenate was filtered through cheesecloth to remove coarse particles before titration. A 25 mL aliquot of the filtrate was titrated with standardized 0.1 M NaOH to an endpoint of pH 8.1 using a calibrated digital pH meter. The NaOH solution was standardized against potassium hydrogen phthalate before use. All titrations were performed at room temperature (25 °C) in triplicate. Titratable acidity was expressed as lactic acid equivalents and calculated using Equation (1):

(1) $$\mathrm{TA}\%=\frac{V\times M\times 90.08\times \mathrm{DF}}{W}$$

where W is the sample weight (g), DF is the dilution factor, V is the volume of NaOH used (mL), M is the molarity of NaOH (mol/L), and 90.08 is the molecular weight of lactic acid (g/mol). The findings were expressed as g of lactic acid per 100 g of fresh pumpkin weight.

#### 2.4.3. Determination of Reducing Sugar

Reducing sugars were determined using the 3,5-dinitrosalicylic acid (DNS) method according to Miller [20] with minor modifications. At each fermentation time point, pumpkin puree samples were centrifuged at 4500× *g* for 15 min at 4 °C, and the supernatant was collected for analysis. The supernatant was diluted with distilled water using a fixed dilution factor (e.g., 5-fold or 10-fold) to ensure that absorbance values fell within the linear range of the glucose standard curve. Then, 1.5 mL of diluted sample was mixed with 1.5 mL of DNS reagent and heated in a boiling water bath for exactly 5 min to allow color development. The reaction was immediately stopped by cooling the tubes in an ice bath. Absorbance was measured at 540 nm using a UV-Vis spectrophotometer (UV-1800, Shimadzu, Kyoto, Japan). A reagent blank was prepared using distilled water instead of the sample. A glucose standard curve ranging from 0 to 1.0 mg/mL was prepared and treated under the same conditions as the samples, with R^2^ > 0.99. All measurements were performed in triplicate. Reducing sugar concentrations were calculated from the glucose calibration curve and expressed as g glucose equivalents per liter of pumpkin extract.

#### 2.4.4. Determination of Total Soluble Sugars

The quantification of soluble sugars in pumpkin puree samples was performed using high-performance liquid chromatography (HPLC) according to the previously described method [16] with minor modifications. Briefly, pumpkin puree samples were centrifuged at 10,000 rpm for 10 min at 4 °C using a refrigerated centrifuge (CR21GIII, Hitachi, Japan) to remove particulate matter. The supernatant was collected and filtered through a 0.45 μm polyethersulfone membrane filter before transfer into HPLC vials. Chromatographic analysis was performed using an HPLC system (Shimadzu, Japan) equipped with a refractive index detector (RID). Separation was carried out using a reverse-phase C18 column, such as the ZORBAX Eclipse Plus C18 column (4.6 × 250 mm, 5 μm particle size, 95 Å pore size Agilent Technologies, Santa Clara, CA, USA). The mobile phase consisted of 0.1 M KH_2_PO_4_–H_3_PO_4_ buffer solution, pH 6.7, and acetonitrile at a ratio of 83:17 (*v*/*v*). The system was operated under isocratic conditions at a flow rate of 1.0 mL/min. The injection volume was 20 μL, the column temperature was maintained at 30 °C, and the RID temperature was maintained at 35 °C. The total analysis time was 30 min per sample. Glucose, fructose, sucrose, and maltose were identified by comparing their retention times with authentic standards. Quantification was performed using external calibration curves prepared from standard solutions of glucose, fructose, sucrose, and maltose, and all calibration curves showed good linearity with R^2^ > 0.99 [21].

### 2.5. Determination of Bioactive Compounds

#### 2.5.1. Total Phenolic Content (TPC)

The TPC of fermented pumpkin samples was determined using the Folin–Ciocalteu colorimetric process [22]. Briefly, 0.5 mL of appropriately diluted sample extract was mixed with 2.5 mL of 10% (*v*/*v*) Folin–Ciocalteu reagent. Then, 2.0 mL of sodium carbonate solution (75 g/L) was added, and the mixture was vortexed thoroughly. The reaction mixture was incubated at room temperature in the dark for 40 min to allow color development. After incubation, 200 µL of the reaction mixture was transferred to a 96-well microplate, and absorbance was measured at 765 nm using a Spark 10M microplate reader (Spark, Tecan, Austria). A reagent blank containing distilled water instead of the sample was prepared under the same conditions. Gallic acid was used as the standard, and the calibration curve was prepared over the range of 0–200 mg/L with good linearity (R^2^ > 0.99). All measurements were performed in triplicate, and the results were expressed as milligrams of gallic acid equivalents per gram fresh weight of pumpkin puree (μg GAE/g). Because the Folin–Ciocalteu assay may also react with non-phenolic reducing compounds, the results were interpreted as total reducing capacity expressed as gallic acid equivalents rather than as a direct measurement of individual phenolic compounds.

#### 2.5.2. HPLC Analysis of Phenolic Compounds

High-performance liquid chromatography (HPLC) was used to analyze phenolic compounds according to a previously described method [23] with minor modifications. Samples were centrifuged at 10,000× *g* for 10 min at 4 °C, and the supernatant was filtered through a 0.45 μm PES membrane filter before injection. Chromatographic separation was performed using a ZORBAX Eclipse Plus C18 column (4.6 × 250 mm, 5 μm particle size, 95 Å pore size; Agilent Technologies, Santa Clara, CA, USA). The mobile phase consisted of 1% formic acid in water (solvent A) and acetonitrile (solvent B). The gradient program was: 5% B from 0 to 5 min, 12% B from 5 to 25 min, 30% B from 25 to 40 min, 45% B from 40 to 50 min, and 5% B from 50 to 60 min for column re-equilibration. The flow rate was fixed at 1.0 mL, and the column temperature was maintained at 30 °C. A UV detector set to 280 nm was used to identify phenolic chemicals. All measurements were performed in triplicate. Quantification was carried out using external calibration curves of corresponding standards, and individual compounds were identified by comparing their retention times with those of genuine standards.

### 2.6. Antioxidant Activities

#### 2.6.1. DPPH Radical Scavenging Activity

DPPH radical scavenging activity of pumpkin puree was measured using a slightly modified version of the previously published method [16]. In short, 1.5 mL of 0.2 mM DPPH solution made in 80% (*v*/*v*) ethanol was combined with 0.5 mL of suitably diluted sample extract. After 10 s of vortexing, the mixture was incubated for 30 min at room temperature in the dark. Following incubation, a microplate reader (Spark, Tecan, Austria) was used to measure the absorbance at 517 nm. A sample blank (extract + ethanol without DPPH) was employed to account for the extract’s inherent absorbance, and a control was set up by substituting 80% ethanol for the sample extract. To verify test performance, ascorbic acid (VC) was employed as a positive control. All measurements were performed in triplicate. The equation was used to estimate the DPPH radical-scavenging activity (2):

(2) $$\mathrm{DPPH}\%=\left[1-\frac{A_{sample}-A_{blank}}{A_{control}}\right]\times 100$$

where A blank is the absorbance of the sample without DPPH, A control is the absorbance of the DPPH solution without the sample, and A sample is the absorbance of the reaction mixture comprising the sample and DPPH.

#### 2.6.2. ABTS Radical Scavenging Activity

ABTS radical-scavenging activity of fermented pumpkin extracts was determined according to a previously reported method [16] with minor modifications. Briefly, ABTS radical cation solution was prepared by mixing ABTS solution (7 mmol/L) with potassium persulfate solution (2.45 mmol/L) in equal volumes. The mixture was incubated at room temperature in the dark for 16 h to generate the ABTS^+^ radical cation. Before analysis, the ABTS^+^ stock solution was diluted with 70% ethanol to obtain an absorbance of 0.70 ± 0.02 at 734 nm. Then, 0.4 mL of sample extract was mixed with 3.6 mL of ABTS^+^ working solution and incubated at room temperature in the dark for exactly 6 min. The reaction time was kept constant for all samples and standards to ensure measurement stability and comparability. Absorbance was measured at 734 nm using a microplate reader (Spark, Tecan, Austria). A control was prepared by replacing the sample extract with 70% ethanol, while a sample blank containing extract and ethanol without ABTS^+^ solution was used to correct background absorbance. Ascorbic acid was used as a positive reference compound. All measurements were performed in triplicate. ABTS radical-scavenging activity was calculated using the following equation:

(3) $$\mathrm{ABTS}\%=\left[1-\frac{A_{sample}-A_{blank}}{A_{control}}\right]\times 100$$

where A control is the absorbance of the ABTS solution without the sample, A blank is the absorbance of the sample without ABTS, and A sample is the absorbance of the reaction mixture containing the sample and ABTS.

### 2.7. Enzyme Inhibition Assays

#### 2.7.1. α-Glucosidase Inhibition Assay

Fermented pumpkin extracts’ α-glucosidase inhibitory activity was assessed using a previously described technique [24] with minor alterations. In summary, 100 μL of sample solution and 100 μL of α-glucosidase solution (0.5 U/mL) were combined, and the mixture was incubated for 10 min at 37 °C. The reaction mixture was then incubated at 37 °C for 20 min after 100 μL of 5 mmol/L p-nitrophenyl-α-D-glucopyranoside (pNPG) was added as the substrate. Using a microplate reader (Spark, Tecan, Austria), the absorbance at 405 nm was used to measure the release of p-nitrophenol. As a positive control inhibitor, acarbose (0.1 mg/mL) was employed. A blank was made without the enzyme to account for background absorbance, and a control reaction was set up by substituting the sample with solvent. Equation was used to calculate the percentage of α-glucosidase inhibition (4):

(4) $$\alpha \text{-}\mathrm{glucosidase}\mathrm{inhibition}\left(\%\right)=\left[1-\frac{A_{sample}-A_{blank}}{A_{control}}\right]\times 100$$

In this case, A control is the absorbance of the reaction without the inhibitor, A blank is the absorbance of the reaction mixture without the enzyme, and A sample is the absorbance of the reaction mixture containing both the sample and the enzyme.

#### 2.7.2. α-Amylase Inhibition Assay

α-Amylase inhibitory activity of fermented pumpkin extracts was determined using a previously reported method [24] with minor modifications. Briefly, 100 µL of sample extract was mixed with 100 µL of α-amylase solution (0.2 U/mL, prepared in 20 mM phosphate buffer, pH 6.9) and pre-incubated at 25 °C for 10 min. Then, 200 µL of 1% (*w/v*) soluble starch solution prepared in the same phosphate buffer was added to initiate the reaction, followed by incubation at 25 °C for 10 min. The reaction was stopped by adding 200 µL of DNS reagent, and the mixture was heated in a boiling water bath for exactly 5 min to develop color. DNS reacts with reducing sugars released from starch hydrolysis; therefore, lower absorbance at 540 nm indicates stronger α-amylase inhibition. After cooling to room temperature, the reaction mixture was diluted with distilled water, and absorbance was measured at 540 nm using a microplate reader (Spark, Tecan, Austria). Acarbose (0.1 mg/mL) was used as a positive control. A control reaction was prepared by replacing the sample with solvent, and a sample blank without enzyme was used to correct background absorbance. All measurements were performed in triplicate. α-Amylase inhibitory activity was expressed as percentage inhibition using the following equation:

(5) $$\alpha \text{-}\mathrm{amylase}\mathrm{inhibition}\left(\%\right)=\left[1-\frac{A_{sample}-A_{blank}}{A_{control}}\right]\times 100$$

where A control is the absorbance of the reaction mixture without inhibitor, A blank is the absorbance of the reaction mixture without enzyme, and A sample is the absorbance of the reaction mixture with the sample and enzyme.

### 2.8. Cell Culture and Glucose Uptake Measurement

L6 rat skeletal muscle myoblasts were cultured at 37 °C in a humidified atmosphere containing 5% CO_2_ in low-glucose DMEM (5.5 mM glucose) supplemented with 10% FBS, 100 U/mL penicillin, and 100 µg/mL streptomycin. At approximately 80–90% confluence, differentiation was induced by replacing the growth medium with DMEM containing 2% FBS. The medium was changed every 48 h for 4–6 days until multinucleated myotubes were formed [25]. Insulin resistance was induced by exposing differentiated myotubes to high-glucose DMEM containing 25 mM glucose and 100 nM insulin for 24 h, while normal control cells were maintained in normal-glucose DMEM containing 5.5 mM glucose. Non-fermented and LAB-fermented pumpkin powders were standardized on a dry weight basis. The freeze-dried powders were dissolved in serum-free DMEM, vortexed for 2 min, sonicated for 20 min, and centrifuged at 5000× *g* for 10 min. The supernatants were collected and sterilized using a 0.22 µm membrane filter. Fresh stock solutions were prepared before each experiment, and working concentrations of 10, 50, and 100 µg/mL were expressed as µg dry weight equivalent per mL of culture medium. The concentrations were not normalized to total phenolic content because the aim was to compare the effects of equal dry amounts of pumpkin material after fermentation. Cytotoxicity was estimated by treating myotubes with 0–200 µg/mL pumpkin extracts for 24 h, followed by MTT assay. Concentrations maintaining ≥90% cell viability were selected for further experiments, namely 10, 50, and 100 µg/mL [26]. For glucose uptake experiments, cells were divided into normal glucose control, insulin-resistant control, insulin-resistant cells treated with non-fermented pumpkin extract, insulin-resistant cells treated with fermented pumpkin extract, and insulin-resistant cells treated with metformin as the positive control. Cells were treated for 24 h, and under insulin-stimulated conditions, 100 nM insulin was added during the final 30–60 min before glucose uptake measurement. Glucose uptake was assessed using 2-NBDG by washing cells with PBS, pre-incubating them in glucose-free DMEM for 30 min, incubating with 100 µM 2-NBDG for 20–30 min at 37 °C in the dark, washing three times with ice-cold PBS, and measuring fluorescence at emission wavelengths of 485/535 nm. Results were expressed as RFU or normalized relative to the control group [27].

### 2.9. Statistical Analysis

All results are expressed as mean ± standard deviation (SD) from triplicate measurements. Statistical significance was evaluated using one-way analysis of variance (ANOVA) followed by Tukey’s multiple comparison test, with *p* < 0.05 considered statistically significant. All statistical analyses were performed using SPSS version 26.0 (IBM Corp., Armonk, NY, USA). Pearson’s correlation analysis and heatmap visualization were conducted, and corresponding graphs were generated using Origin 2021 (OriginLab Corp., Northampton, MA, USA).

## 3. Results and Discussion

### 3.1. Phsiochemical Parameters and Viable Counts

Fermentation performance was confirmed by increased viable LAB counts, pH reduction, higher titratable acidity, and reduced sugar levels, indicating active LAB growth, acid production, and carbohydrate utilization in pumpkin puree.

**Figure 1:**
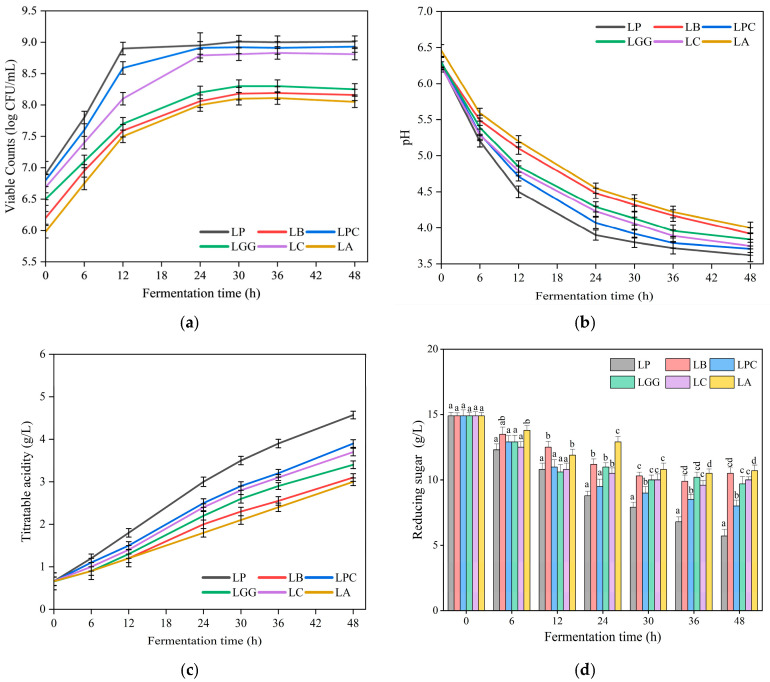
The viable counts (**a**), pH (**b**), titratable acidity (**c**), and reducing sugar (**d**) of pumpkin puree fermented with different LABs, including *Lactobacillus acidophilus* (LA), *Lactobacillus delbrueckii* subsp. *bulgaricus* (LB), *Lacticaseibacillus casei* (LC), *Lacticaseibacillus rhamnosus* GG (LGG), *Lacticaseibacillus paracasei* (LPC), and *Lactiplantibacillus plantarum* (LP). Results are expressed as mean ± standard deviation (n = 3). Different lowercase letters indicate significant differences (*p* < 0.05). Values of the reducing sugar (**d**) at each time interval with different small letters above the error bar are significant (*p* < 0.05).

As shown in Figure 1a, all LAB strains exhibited rapid growth during the first 12 h of fermentation, attaining the exponential phase, after which growth gradually stabilized. LP, LPC, and LC maintained the highest viable counts (~9.0 log CFU/g) at the end of fermentation, whereas LGG, LB, and LA showed slightly lower populations (~7.9–8.2 log CFU/g). The decrease in pH from 6.2 to 6.5 to 3.50–3.61 was associated with organic acid production during carbohydrate metabolism. Concurrently, reducing sugars decreased from 14.93 g/L to 6.51–10.52 g/L, indicating active substrate utilization by LAB [23]. These metabolic changes may contribute to the release or transformation of bound phytochemicals, thereby supporting the enhanced bioactive properties observed in fermented pumpkin puree [16].

As illustrated in Figure 1b, fermentation with LAB resulted in rapid acidification of pumpkin puree, with the pH decreasing from an early value of approximately 6.2–6.5 to 3.50–3.61 after fermentation. This decrease is mainly ascribed to the accumulation of organic acids, mainly lactic acid and minor amounts of acetic acid, generated during the metabolism of soluble sugars naturally present in pumpkin [28]. The most pronounced pH reduction occurred within the first 12 h, corresponding to the exponential growth phase of LAB and intensive substrate utilization. Between 12 and 24 h, the rate of pH declined gradually, slowed as microbial growth approached the stationary phase, and acid accumulation inhibited further metabolic activity [29]. After 24–30 h of fermentation, the pH of all samples dropped below 4.5, indicating effective acidification of the substrate. Among the tested strains, LP exhibited the strongest acidification capacity, producing the lowest final pH, followed by LPC and LC, whereas LGG, LB, and LA showed comparatively weaker acidification ability. These strain-dependent differences in acid production may influence the release and transformation of bioactive compounds in pumpkin puree, which could contribute to the enhanced functional properties observed in fermented products [12].

As shown in Figure 1c, the TA of pumpkin puree increased significantly during fermentation (*p* < 0.05), corresponding to the progressive collection of organic acids generated by lactic acid bacteria (LAB). This increase is mainly attributed to the conversion of soluble sugars in pumpkin, such as glucose, fructose, and sucrose, into lactic acid and minor organic acids through microbial fermentation pathways [30]. Consistent with the pH results, LP- and LPC-fermented samples exhibited the highest TA values, indicating stronger acid-producing capacity and more active carbohydrate metabolism. In contrast, the LA-fermented sample showed the lowest TA value at the end of fermentation, suggesting relatively weaker metabolic activity of this strain in the pumpkin substrate. The strain-dependent differences in acid production may influence the biochemical transformation of pumpkin components during fermentation, potentially affecting the release and bioavailability of functional compounds associated with health-promoting properties [31,32].

As shown in Figure 1d, the concentration of reducing sugars decreased significantly during fermentation, declining from 14.93 g/L to 6.51–10.52 g/L (*p* < 0.05). The reduction in soluble sugars during fermentation may contribute to lowering the potential glycemic impact of pumpkin puree, which is beneficial for the development of functional foods targeting glucose regulation [33]. Among the tested strains, LP and LPC exhibited the greatest sugar consumption, resulting in the lowest residual sugar concentrations after 48 h of fermentation, followed by LC. In contrast, the LA, LGG, and LB-fermented samples retained relatively higher levels of reducing sugars, signifying comparatively lower substrate utilization under the same conditions. Variances in metabolic activity and adaptability to the pumpkin matrix, which can affect microbial growth and acid production during fermentation, may be linked to these strain-dependent variances in sugar consumption [20].

### 3.2. Total Soluble Sugars

**Figure 2:**
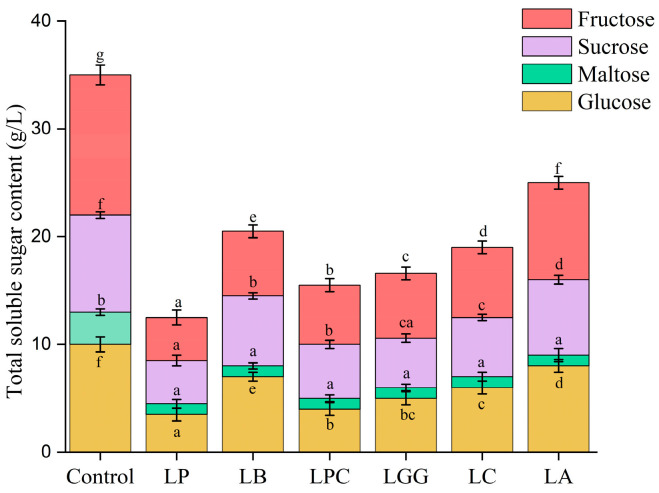
Effect of lactic acid bacteria (LAB) fermentation on soluble sugar composition (glucose, maltose, sucrose, and fructose) of pumpkin puree. Values are expressed as mean ± standard deviation (n = 3). Different small letters in the figure bars indicate significant differences among treatments (*p* < 0.05).

The total soluble sugar content of pumpkin puree before and after LAB fermentation is shown in Figure 2. Soluble sugars are important carbon sources for LAB growth and metabolism, and their reduction during fermentation reflects microbial utilization of available carbohydrates [31]. In the non-fermented control, the total soluble sugar content was approximately 35 g/L, indicating that fresh pumpkin puree contained considerable amounts of fermentable sugars. Fructose, glucose, maltose, and sucrose were the main soluble sugars detected in the control sample, with fructose contributing the largest proportion, followed by glucose, maltose, and sucrose.

After LAB fermentation, total soluble sugar content decreased significantly (*p* < 0.05) in all fermented samples, confirming active carbohydrate consumption by the LAB strains. However, the extent of sugar utilization differed among strains. LP showed the lowest residual total soluble sugar content, approximately 12–13 g/L, indicating the strongest carbohydrate utilization. This was followed by LPC (15–16 g/L), LGG (16–17 g/L), and LC (~19 g/L). LB retained comparatively higher residual sugars (20–21 g/L), whereas LA showed the highest remaining sugar content among the fermented samples (~25 g/L), suggesting relatively weaker substrate utilization. These results indicate that sugar metabolism during fermentation was strain-dependent, with LP showing the strongest adaptation to the pumpkin puree matrix.

Analysis of individual sugars further confirmed the different sugar utilization patterns among LAB strains. Glucose was one of the major sugars in the control sample, with an initial concentration of approximately 10 g/L. After fermentation, glucose decreased markedly, reaching the lowest level in LP-fermented pumpkin (~3.5 g/L), while LA retained a comparatively higher glucose level (~8 g/L) [34]. This reduction suggests that glucose served as a primary carbon source for LAB growth and organic acid production. Maltose also decreased after fermentation, from approximately 9 g/L in the control to about 4–7 g/L in fermented samples, with the lowest levels observed in LP and LPC. Sucrose showed a similar decreasing trend, declining from approximately 3 g/L in the control to about 1–2 g/L after fermentation, possibly due to microbial hydrolysis followed by utilization of the resulting monosaccharides. Fructose was the most abundant sugar in the control sample, contributing approximately 13 g/L, but it also decreased noticeably after fermentation [14].

The greater depletion of soluble sugars in LP-fermented pumpkin puree agrees with its higher viable cell count and stronger acidification capacity, suggesting more efficient carbohydrate metabolism. In contrast, the higher residual sugar content in LA-fermented samples may be related to slower growth, selective sugar metabolism, or lower enzymatic activity in the pumpkin puree matrix. The reduction in soluble sugars was also consistent with the observed decrease in pH and increase in titratable acidity, indicating conversion of fermentable sugars into organic acids, particularly lactic acids. From a functional perspective, reduced simple sugar levels may contribute to a lower glycemic load, while organic acid production may improve preservation and support the development of fermented pumpkin puree as a functional food with potential relevance for glycemic management [35]. Additionally, reduced simple sugars may be beneficial for glycemic control, supporting the development of functional foods targeting diabetes management [36].

### 3.3. Bioactive Compounds

**Figure 3:**
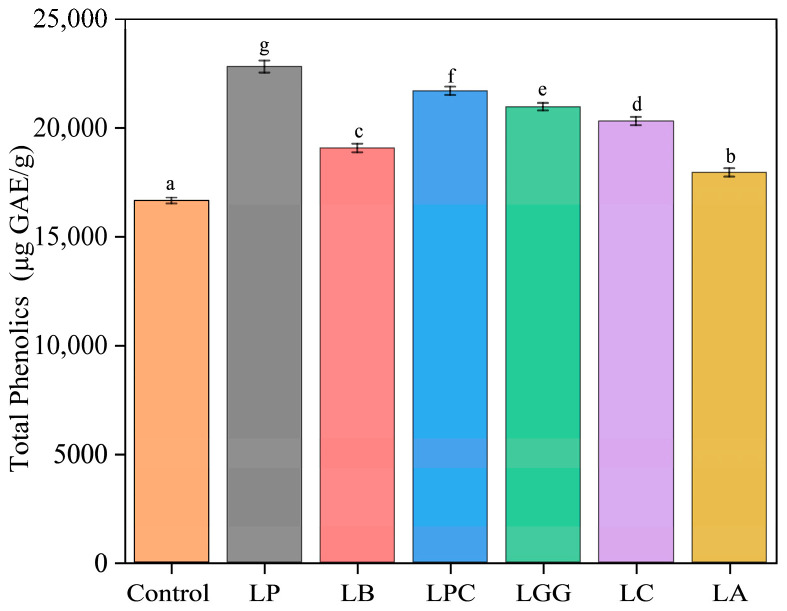
Total phenolic content (TPC) of LAB-fermented pumpkin samples. The TPC was determined using the Folin–Ciocalteu colorimetric method (expressed as μg GAE/g) for each LAB strain: LP, LB, LPC, LGG, LC, LA, and the unfermented Control. Values are presented as mean ± SD (n = 3). Different letters above the bars indicate significant differences between strains (*p* < 0.05).

Important bioactive components of fruits and vegetables, phenolic compounds have been linked to several health advantages, such as anti-inflammatory, anticancer, and antioxidant properties [37]. As shown in Figure 3, following fermentation with LAB, the TPC of pumpkin dramatically increased. Significant variations between strains are indicated by different letters above the bars (*p* < 0.05). The control that was not fermented displayed the lowest FC-TPC (~17,632 µg GAE/g), while all fermented samples showed higher values (~19,018–23,363 µg GAE/g), following the order: LP (~23,363 µg GAE/g) > LPC (~22,702 µg GAE/g) > LGG (~21,757 µg GAE/g) > LC (~20,686 µg GAE/g) > LB (~20,057 µg GAE/g) > LA (~19,018 µg GAE/g). The increase in TPC after fermentation is consistent with previous reports indicating that LAB fermentation enhances extractable phenolics through acidification-induced cell wall disruption and enzymatic hydrolysis, which release bound phenolic compounds and convert conjugated forms into more soluble and detectable molecules. These transformations are often strain dependent [38].

The phenolic composition of pumpkin was also significantly modified following fermentation with different LAB strains (Table 1, *p* < 0.05). HPLC analysis revealed substantial increases in several individual phenolic acids and flavonoids in fermented samples, with LP generally producing the highest concentrations. Amongst the quantified phenolics, trans-ferulic acid was the predominant compound, increasing from 2506.20 ± 15.25 in the control sample to 3894.29 ± 1.25 in LP-fermented pumpkin. Elevated levels were also observed in LPC (3732.22 ± 14.20), LGG (3581.24 ± 9.99), and LA (3425.91 ± 13.61). This increase can be clarified by the structural role of ferulic acid in plant cell walls, where it usually occurs in ester-linked forms associated with arabinoxylans and participates in cross-linking with polysaccharides and lignin. During fermentation, phenolic esterases produced by LAB can analyze these ester bonds, releasing free ferulic acid that becomes more readily detectable by chromatographic analysis [38].

Similarly, several other phenolic acids increased substantially after fermentation, including gallic acid (from 1143.94 ± 4.60 to 1996.32 ± 7.27 μg/g), 3,4-dihydroxybenzoic acid (from 1244.75 ± 5.78 to 1855.24 ± 5.03 μg/g), caffeic acid (from 1324.66 ± 2.09 to 1894.16 ± 4.13 μg/g), and syringic acid (from 1445.60 ± 9.55 to 1860.77 ± 8.30 μg/g), suggesting that fermentation promotes both the release of bound phenolics and the biotransformation of phenolic compounds through strain-dependent metabolic activity [39]. In addition to phenolic acids, flavonoid-related compounds also increased following fermentation. For example, vitexin (from 128.68 ± 2.48 to 181.88 ± 2.91 μg/g), quercetin-3-β-D-glucoside (from 111.65 ± 3.40 to 182.66 ± 3.22 μg/g), and luteoloside (from 119.70 ± 1.21 to 174.99 ± 1.90 μg/g) increased in LP-fermented samples compared with the control. These results agree with previous studies reporting that LAB fermentation can enhance phenolic extractability and modify glycosylated flavonoids through enzymatic activities such as β-glucosidase, which convert bound phenolics into more bioavailable forms. Notably, these transformations are strongly influenced by strain-specific enzymatic systems [40].

### 3.4. Antioxidant properties

**Figure 4:**
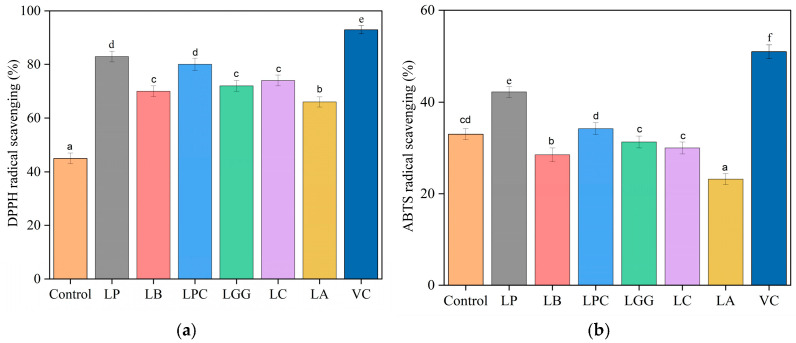
DPPH (**a**) and ABTS (**b**) radical-scavenging activities of pumpkin puree after 48 h of in vitro fermentation with different LAB strains, including *Lactobacillus acidophilus* (LA), *Lactobacillus delbrueckii* subsp. *bulgaricus* (LB), *Lacticaseibacillus casei* (LC), *Lacticaseibacillus rhamnosus* GG (LGG), *Lacticaseibacillus paracasei* (LPC), and *Lactiplantibacillus plantarum* (LP) compared with the non-inoculated control and VC (ascorbic acid, positive control, specified concentration) was included to validate assay performance. Results are expressed as mean ± standard deviation (n = 3). Different lowercase letters above the column bars indicate significant differences (*p* < 0.05).

DPPH and ABTS radical scavenging assays showed that LAB fermentation improved the antioxidant potential of pumpkin puree (Figure 4a,b), but the magnitude of improvement was strain- and assay-dependent rather than uniform across all samples. In the DPPH assay, the strongest response was observed for LP, where radical scavenging activity increased from approximately 45% in the non-fermented control to 83.2%, representing an increase of 38.2% points and an approximate 85% relative improvement. LPC also showed a strong increase to 80.4%, followed by LC, LGG, LB, and LA. This pattern suggests that *Lactiplantibacillus plantarum* and *Lacticaseibacillus paracasei* were more effective in enhancing the antioxidant potential of pumpkin puree than the other tested strains. Instead of simply reflecting microbial growth, this improvement may be associated with strain-specific enzymatic activities, including β-glucosidase, esterase, and phenolic acid decarboxylase activities, which can release bound phenolics from the plant matrix and convert them into compounds with higher radical-scavenging capacity [29]. Compared with previous pumpkin-based fermentation studies, the antioxidant enhancement observed here was substantial but not as pronounced as that reported by earlier studies [16] Sun et al. (2022) in LAB-fermented pumpkin juice. Fermented pumpkin juice with five LAB strains at 37 °C for 48 h and reported significant improvement in DPPH and hydroxyl radical scavenging capacities, with stronger responses particularly in *L. plantarum*, *L. acidophilus*, and *L. helveticus* fermented samples. They further associated the antioxidant improvement with fermentation-induced changes in phenolic profiles, especially vanillic acid and sinapic acid. All LAB-fermented samples showed significantly higher antioxidant capacities than the control, with clear strain-dependent differences [41]. In the DPPH assay, LP exhibited the highest radical scavenging activity (83.2%), followed by LPC (80.4%), LC (74.0%), LGG (72.3%), and LB (70.0%). Despite being significantly greater than the control (*p* < 0.05), LA exhibited the lowest activity among the fermented samples (66.2%).

A typically comparable trend was detected in the ABTS assay, although the absolute scavenging values differed. LP again exposed the strongest activity (42.23%), followed by LPC (34.2%), LGG (31.2%), and LC (30.0%). LB exhibited moderate activity (28.5%), while LA showed the lowest activity (23.2%), which did not significantly differ from the control (*p* > 0.05). As a positive control, ascorbic acid (VC) showed the greatest capacity to scavenge radicals (DPPH ≈ 93%, ABTS ≈ 51%). The enhanced antioxidant activity observed in LP- and LPC-fermented samples may be linked with their greater ability to release and transform bioactive compounds during fermentation [42]. ABTS showed a narrower and strain-dependent response, with LP showing the clearest improvement. Therefore, unlike DPPH activity, ABTS scavenging activity did not increase uniformly in all fermented samples. LAB enzymatic activities, such as β-glucosidase and esterase, can hydrolyze bound phenolics from plant cell wall matrices, thus increasing the availability of antioxidant compounds. Moreover, fermentation metabolism may generate other antioxidant molecules, including organic acids, peptides, and small phenolic metabolites. These biochemical transformations are often strain dependent and contribute to the observed differences in antioxidant capacity [43]. The differences among DPPH and ABTS results may be ascribed to the distinct chemical properties of the two radicals and their sensitivity to different antioxidant compounds [44]. DPPH is generally receptive to lipophilic antioxidants, whereas ABTS can react with both hydrophilic and lipophilic antioxidants, which may clarify the variation in absolute scavenging values. Inclusive, these findings indicate that LAB fermentation, particularly with strains such as LP and LPC, can significantly enhance the antioxidant potential of pumpkin puree in a strain-dependent manner [14]. Though it should be stated that in vitro antioxidant assays do not certainly reflect in vivo antioxidant effects, further studies are required to prove their physiological relevance.

### 3.5. α-Glucosidase and α-Amylase Inhibition

**Figure 5:**
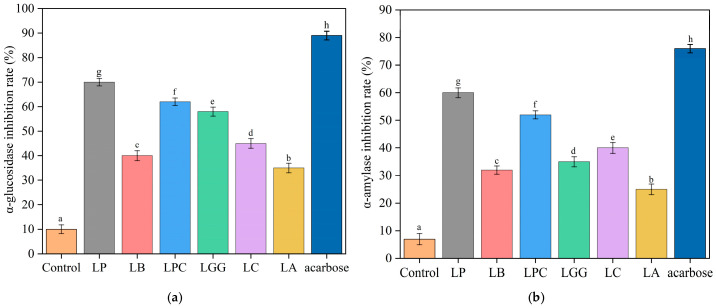
α-glucosidase (**a**) and α-amylase (**b**) inhibition activities of pumpkin puree after 48 h of in vitro fermentation with different LAB strains, including *Lactobacillus acidophilus* (LA), *Lactobacillus delbrueckii* subsp. *bulgaricus* (LB), *Lacticaseibacillus casei* (LC), *Lacticaseibacillus rhamnosus* GG (LGG), *Lacticaseibacillus paracasei* (LPC), and *Lactiplantibacillus plantarum* (LP) compared with the non-inoculated control. Results are expressed as mean ± standard deviation (n = 3). Different lowercase letters above the column bars indicate significant differences (*p* < 0.05).

The digestive enzymes α-glucosidase and α-amylase play a significant role in the metabolism of carbohydrates. Starch is first hydrolyzed by α-amylase into oligosaccharides, which are then further hydrolyzed into glucose in the small intestine by α-glucosidase. In people with type II diabetes, inhibition of these enzymes can lower postprandial blood glucose levels by slowing the digestion of carbohydrates and the absorption of glucose [45]. So, the inhibitory effects of six LAB-fermented pumpkin samples on α-glucosidase and α-amylase were assessed to classify strains with potential hypoglycemic activity (Figure 5a,b). LAB fermentation significantly increased α-glucosidase inhibitory activity compared with the unfermented control (approximately 10%). The inhibition levels ranged from 35% to 70%, signifying clear strain-dependent differences. Among the tested strains, LP showed the strongest inhibition (70%), followed by LPC (62%), LGG (58%), and LC (45%), while LB (40%) and LA (35%) exhibited practically lower inhibitory activity. Acarbose (0.1 mg/mL), used as a positive control, showed the peak inhibition (89%), confirming the validity of the assay and indicating that several fermented samples exhibited significant in vitro inhibitory activity relative to the pharmaceutical reference. The observed strain-specific variations could be explained by LAB-mediated alteration of the plant matrix during fermentation, which can improve phenolic compound release and biotransformation and produce bioactive metabolites like organic acids and peptides, influencing enzyme inhibition based on each strain’s metabolic capacities [46].

A similar strain-dependent trend was observed in the α-amylase inhibition assay. The unfermented control exhibited low inhibition (approximately 7%), whereas LAB fermentation markedly increased inhibitory activity. LP again demonstrated the strongest inhibition (60.2%), followed by LPC (52.3%), LC (40.1%), and LGG (35.2%), while LB (32.2%) and LA (25.3%) showed lower inhibition levels. Acarbose exhibited the highest inhibitory activity (76%), consistent with its well-known pharmacological effect. These results are consistent with prior studies reporting that LAB fermentation can enhance α-amylase and α-glucosidase inhibition in fruit and vegetable matrices through increased release or transformation of phenolic compounds and the production of low-molecular-weight inhibitory metabolites such as organic acids and bioactive peptides [46]. Overall, fermentation improved the enzyme-inhibitory potential of pumpkin puree, with LP showing the strongest hypoglycemic-related activity among the tested strains. Though it should be distinguished that these outcomes are based on in vitro assays, and further in vivo studies, including simulated gastrointestinal digestion to verify the physiological significance and possible antidiabetic effects of fermented pumpkin products, studies are required [47]. The increased α-amylase and α-glucosidase inhibition suggests that LAB-fermented pumpkin puree may help slow carbohydrate digestion and glucose release. However, these in vitro results are preliminary and do not confirm actual anti-diabetic effects in vivo. Further simulated digestion, animal, and human studies are needed to validate its physiological relevance.

### 3.6. Cell Culture Glucose Uptake

**Figure 6:**
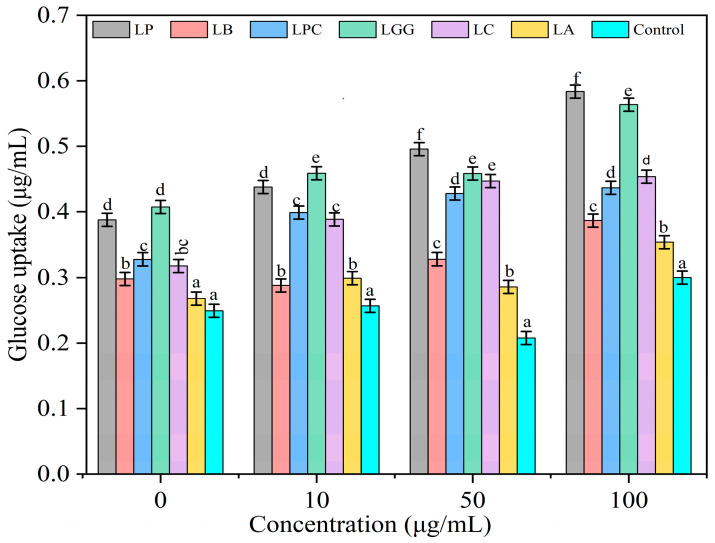
Effects of LAB-fermented pumpkin puree extracts on insulin-stimulated glucose uptake in insulin-resistant L6 myotubes. Cells were treated for 24 h with pumpkin extracts fermented by LAB strains, including *Lactobacillus acidophilus* (LA), *Lactobacillus delbrueckii* subsp. *bulgaricus* (LB), *Lacticaseibacillus casei* (LC), *Lacticaseibacillus rhamnosus* GG (LGG), *Lacticaseibacillus paracasei* (LPC), and *Lactiplantibacillus plantarum* (LP) at 0, 10, 50, and 100 μg/mL, followed by insulin stimulation (100 nM) and measurement of 2-NBDG uptake by flow cytometry (fluorescence intensity). Control represents the insulin-resistant model treated with vehicle (no extract). Data are presented as mean ± SD (n = 3). Different lowercase letters above bars within the same concentration indicate significant differences (*p* < 0.05).

As shown in Figure 6, glucose uptake increased in a concentration-dependent manner across all treatments. At baseline (0 µg/mL), glucose uptake ranged from 0.25 to 0.41 µg/mL, with LGG (0.41 µg/mL) and LP (0.39 µg/mL) showing higher uptake than the control (0.25 µg/mL). At 10 µg/mL, LP (0.44 µg/mL) and LGG (0.46 µg/mL) maintained the highest uptake levels, whereas the control remained relatively low (0.26 µg/mL). At 50 µg/mL, glucose uptake further increased, with LP reaching approximately 0.50 µg/mL, while LGG, LPC, and LC remained elevated (0.43–0.46 µg/mL), and the control exhibited the lowest uptake (0.21 µg/mL). The maximal effect was observed at 100 µg/mL, where LP (0.58 µg/mL) and LGG (0.56 µg/mL) showed the strongest stimulation of glucose uptake, followed by LC (0.46 µg/mL) and LPC (0.44 µg/mL). LB (0.39 µg/mL) and LA (0.35 µg/mL) exhibited moderate increases, while the control reached approximately 0.30 µg/mL. The improved glucose uptake may be associated with fermentation-induced changes in pumpkin bioactive compounds, including phenolics, organic acids, and other metabolites. However, since this study measured only glucose uptake and did not assess AMPK, Akt, or GLUT4 signaling, the results should be interpreted as functional evidence rather than direct mechanistic proof. Thus, AMPK activation and GLUT4 translocation are considered possible mechanisms that require further confirmation [25]. Previous studies have shown that LAB fermentation can transform phenolic profiles through enzymatic activities such as glycosidase and decarboxylase, release bound phenolics from plant matrices, and improve antioxidant activity and bioavailability. However, because the present study did not identify the exact metabolites responsible for the glucose uptake-promoting effect, these findings should be interpreted as associations rather than confirmed mechanisms. Therefore, future LC–MS/MS metabolomics, simulated gastrointestinal digestion, phenolic bioaccessibility analysis, and validation with purified compounds are needed to confirm the active compounds and their biological relevance [42].

### 3.7. Heat Map

**Figure 7:**
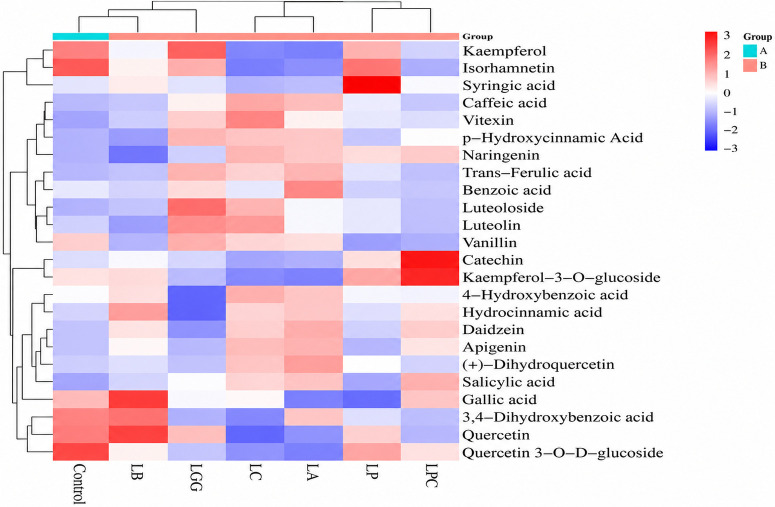
Heat map evaluating the volatile compounds of pumpkin by LAB fermentation.

As illustrated in Figure 7, the heatmap analysis revealed diverse variations in the phenolic composition among the six LAB strains (LP, LB, LPC, LGG, LC, and LA) and the unfermented control indicated a strong strain-dependent effect of fermentation on bioactive compound profiles. Among the tested strains, LP and LPC exhibited relatively higher levels of various phenolic compounds, including kaempferol, caffeic acid, and trans-ferulic acid. This enhancement suggests that these strains may enhance the phenolic profile through microbial biotransformation processes, such as enzymatic hydrolysis of bound phenolics mediated by β-glucosidase and esterase activities [48]. In disparity, LB and LA showed comparatively lower levels of several phenolic constituents, which may reflect differences in strain-specific metabolic pathways and enzymatic capabilities during fermentation. The presence of hydroxycinnamic acids, specifically trans-ferulic acid and p-hydroxycinnamic acid, in samples fermented with LP and LPC provides more evidence for the function of LAB in releasing bound phenolic acids from the food matrix, potentially improving antioxidant efficacy [49]. Moreover, elevated levels of flavonoid glycosides such as vitexin and luteoloside were applied in these strains, suggesting their potential contribution to the improved antioxidant potential of the fermented product. These findings are consistent with previous studies, signifying that LAB fermentation can improve the bioavailability and antioxidant capacity of phenolic compounds in plant-based substrates, complete enzymatic bioconversion, and structural modification of phenolic compounds [50]. In contrast, the unfermented control exhibited markedly lower levels of most bioactive phenolics, confirming that microbial fermentation shows a substantial role in modulating the phenolic composition and functional assets of the substrate.

### 3.8. Pearson’s Correlation

**Figure 8:**
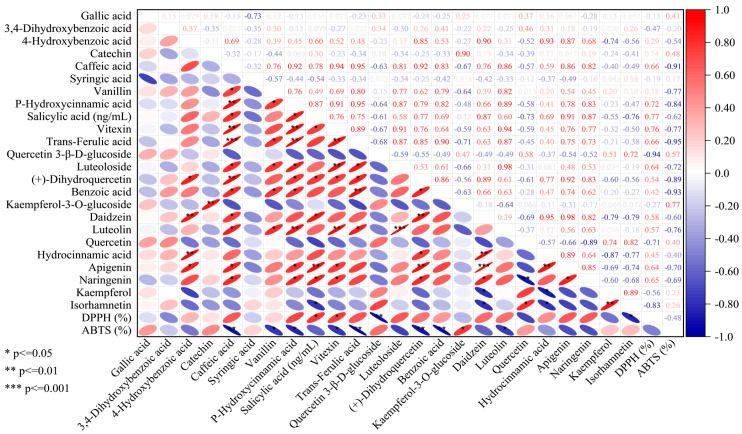
Pearson’s correlation coefficients for phenolic profiles and antioxidant capacities of pumpkin by LAB fermentation.

Phenolic compounds can give reactive radicals, hydrogen atoms, or electrons. They are widely known for being efficient at reducing agents and free radical scavengers [51,52]. To further examine the role of phenolics in the antioxidant potential of LAB-fermented pumpkin puree, Pearson correlation analysis was conducted between TPC and antioxidant activities (Figure 8). A strong positive correlation was observed between TPC and both DPPH (r = 0.91, *p* < 0.05) and ABTS radical scavenging activity (r = 0.89, *p* < 0.05), demonstrating that phenolic molecules are essential to the fermented samples’ overall antioxidant capacity. Among individual phenolics, gallic acid, caffeic acid, and quercetin showed positive correlations with DPPH (r = 0.53, 0.66, and 0.52, respectively) and ABTS (r = 0.61, 0.59, and 0.74, respectively) radical scavenging activities, supporting their well-documented antioxidant properties [53]. Additionally, ferulic acid and DPPH showed a somewhat positive connection activity (r = 0.45), suggesting its contribution to radical scavenging capacity. In contrast, sinapic acid, 3,4-dihydroxybenzoic acid, and coumaric acid displayed negative correlations with both DPPH (r = −0.42, −0.49, and −0.44, respectively) and ABTS (r = −0.47, −0.56, and −0.51, respectively) activities. These negative associations may reflect differences in phenolic composition, relative concentrations, or possible interactions among phenolic compounds within the complex fermented matrix rather than indicating a lack of antioxidant activity [54]. Overall, the correlation analysis suggests that specific phenolic compounds generated or enhanced during LAB fermentation contribute differentially to the antioxidant capacity of fermented pumpkin puree.

## 4. Conclusions

This study demonstrated that LAB fermentation significantly improves the functional and bioactive properties of pumpkin puree. Fermentation resulted in notable, strain-dependent modifications in phenolic composition, antioxidant capacity, and glucose uptake activity. Among the tested strains, *Lactiplantibacillus plantarum* (LP) and *Lacticaseibacillus rhamnosus* GG (LGG) exhibited superior performance, showing higher antioxidant activity, increased phenolic compound availability, and enhanced cellular glucose uptake. Notably, LP demonstrated the most consistent and superior performance, particularly in acidification, antioxidant enhancement, α-amylase and α-glucosidase inhibition, and overall functional improvement. *Lacticaseibacillus paracasei* (LPC) also showed strong effects, especially in promoting glucose uptake in insulin-resistant L6 myotubes; however, its performance was not consistently higher than LP across all measured parameters. Therefore, LP can be considered the most effective strain in the present study, while LPC may be regarded as a promising candidate for improving cellular glucose uptake. In addition, heatmap analysis revealed clear alterations in the phenolic profile after fermentation, with certain LAB-fermented samples showing higher concentrations of key compounds such as caffeic acid, trans-ferulic acid, and kaempferol. These changes are likely associated with microbial biotransformation processes, including enzymatic hydrolysis and the release of bound phenolics. Correlation analysis demonstrated strong positive relationships between total phenolic content and antioxidant activity, highlighting the critical role of phenolic compounds in the functional properties of fermented pumpkin puree. Moreover, individual phenolics, including gallic acid, caffeic acid, and quercetin, were positively correlated with radical scavenging activity, further supporting their antioxidant potential. Overall, these findings suggest that LAB fermentation is an effective strategy to enhance the functional and nutritional value of pumpkin-based foods.

However, several limitations should be addressed in future research: (1) in vivo animal and human intervention studies are needed to validate the observed effects; (2) simulated gastrointestinal digestion studies are required to assess phenolic bioaccessibility; and (3) mechanistic investigations, including GLUT4 translocation imaging and AMPK phosphorylation assays, are necessary to elucidate underlying pathways.

## Figures and Tables

**Table 1:** Polyphenol compounds of pumpkin fermented with different bacteria strains (μg/g).

Phenolic Compounds	LP	LB	LPC	LGG	LC	LA	Control
Gallic acid	1996.32 ± 7.27a	1356.60 ± 8.03d	1728.69 ± 21.85b	1657.57 ± 7.66c	1647.63 ± 1.99c	1296.90 ± 1.85d	1143.94 ± 4.60e
3,4-Dihydroxybenzoic acid	1855.24 ± 5.03a	1325.58 ± 0.78e	1751.44 ± 16.75b	1684.29 ± 6.45c	1648.10 ± 2.67c	1396.69 ± 1.64d	1244.75 ± 5.78f
4-Hydroxybenzoic acid	1780.47 ± 19.78a	1391.65 ± 1.55d	1707.10 ± 3.04a	1610.55 ± 9.63b	1594.41 ± 2.10b	1494.47 ± 4.51c	1306.85 ± 4.00e
Catechin	175.59 ± 4.54a	137.61 ± 2.20c	169.01 ± 0.98a	162.46 ± 2.48b	155.08 ± 2.00b	144.04 ± 1.57c	129.54 ± 3.56d
Caffeic acid	1894.16 ± 4.13a	1357.39 ± 9.00e	1745.71 ± 32.29bc	1709.05 ± 6.06bc	1695.54 ± 2.66c	1455.52 ± 3.02d	1324.66 ± 2.09e
Syringic acid	1860.77 ± 5.05a	1507.14 ± 4.38d	1745.08 ± 10.41bc	1723.50 ± 2.40c	1713.91 ± 2.09c	1545.52 ± 2.90d	1445.60 ± 7.65d
Vanillin	1792.07 ± 7.24a	1456.51 ± 11.71d	1734.33 ± 4.19a	1636.81 ± 3.84b	1626.15 ± 2.64b	1548.55 ± 2.57c	1361.59 ± 9.95e
p-Hydroxycinnamic acid	1645.75 ± 5.02a	1352.84 ± 3.7d	1604.23 ± 2.95a	1595.97 ± 3.41ab	1541.00 ± 3.70bc	1494.68 ± 2.50c	1301.06 ± 1.67d
Salicylic acid	176.21 ± 3.15a	126.51 ± 3.14d	162.55 ± 0.66b	151.10 ± 2.03c	146.09 ± 2.66c	132.55 ± 2.37d	118.40 ± 2.92e
Vitexin	181.88 ± 2.91a	133.06 ± 1.65ef	158.22 ± 1.47b	147.69 ± 1.97c	142.99 ± 2.08cd	139.73 ± 0.90de	128.68 ± 2.48f
Trans-ferulic acid	3894.29 ± 1.25a	2999.29 ± 4.71d	3732.22 ± 14.20a	3581.24 ± 9.99a	3215.39 ± 13.24c	3425.91 ± 13.61b	2506.20 ± 15.25e
Quercetin-3-β-D-glucoside	182.66 ± 3.22a	123.15 ± 2.62e	162.89 ± 2.62b	149.61 ± 2.49c	148.87 ± 1.46c	134.81 ± 4.40d	111.65 ± 3.40f
Luteoloside	174.99 ± 1.90a	128.24 ± 2.47c	167.32 ± 1.42a	156.63 ± 1.52b	148.69 ± 2.22b	134.89 ± 3.12c	119.70 ± 1.21d
(+)-Dihydroquercetin	170.72 ± 2.58a	136.34 ± 4.15c	165.76 ± 2.67a	164.74 ± 2.56a	155.80 ± 2.07b	152.29 ± 3.87b	130.40 ± 3.45c
Benzoic acid	1764.91 ± 3.98a	1484.82 ± 17.04c	1746.65 ± 35.11a	1707.20 ± 4.43a	1695.58 ± 2.51a	1576.46 ± 11.00b	1444.33 ± 5.40c
Kaempferol-3-O-glucoside	168.52 ± 0.64a	137.52 ± 1.53cd	165.06 ± 2.28a	159.10 ± 1.90ab	154.16 ± 2.69b	144.78 ± 4.84c	129.01 ± 1.89d
Daidzein	181.99 ± 2.81a	147.53 ± 1.43c	177.50 ± 1.84a	163.72 ± 1.69b	164.81 ± 1.50b	156.89 ± 1.30b	136.78 ± 2.72d
Luteolin	172.33 ± 1.65a	135.85 ± 2.73d	165.81 ± 1.59ab	157.50 ± 1.98bc	154.34 ± 2.06bcd	146.70 ± 2.57d	128.45 ± 2.34e
Quercetin	166.36 ± 2.41a	148.06 ± 2.46bc	164.28 ± 0.52a	162.58 ± 2.53a	163.70 ± 2.16a	153.65 ± 2.21b	144.54 ± 1.42c
Hydrocinnamic acid	1855.03 ± 8.85a	1705.19 ± 4.17c	1832.75 ± 10.49ab	1793.33 ± 7.11ab	1772.85 ± 6.41ab	1740.28 ± 4.99bc	1693.27 ± 3.20c
Apigenin	182.32 ± 2.58a	153.91 ± 2.08c	177.78 ± 1.51a	177.65 ± 2.16a	168.06 ± 1.85b	155.81 ± 3.49c	147.71 ± 2.79c
Naringenin	190.41 ± 2.29a	164.80 ± 2.39c	186.99 ± 0.26a	185.71 ± 1.89a	181.75 ± 1.38ab	175.15 ± 4.67b	158.78 ± 2.63c
Kaempferol	174.14 ± 1.61a	165.63 ± 2.68ab	165.63 ± 3.66ab	159.37 ± 1.55bc	135.70 ± 3.11d	152.21 ± 2.42c	143.30 ± 2.10d
Isorhamnetin	176.52 ± 2.13a	166.34 ± 2.11b	165.47 ± 2.10b	164.95 ± 2.52b	134.98 ± 2.65d	153.88 ± 1.90c	142.40 ± 1.79d

Values are average of triple measurements with standard deviation. Means in each column with superscripted different small letters are significant (*p* < 0.05).

## Data Availability

The original contributions presented in this study are included in the article. Further inquiries can be directed to the corresponding author.

## Abbreviations

LAB, lactic acid bacteria; DM, diabetes mellitus; LP, *lactiplantibacillus plantarum*; LB, *lactobacillus delbrueckii* subsp. *bulgaricus*; LPC, *lacticaseibacillus paracasei*; LGG, *lacticaseibacillus rhamnosus* GG; LC, *lacticaseibacillus casei*; LA, *lactobacillus acidophilus*; DNS, 3,5-dinitrosalicylic acid; DPPH, 2,2-diphenyl-1-picrylhydrazyl; ABTS, 2,2-azino-bis (3 ethylbenzothiazoline-6-sulfonic acid; CFU, colony forming unit; TTA, total titratable acidity; AMPK, AMP-activated protein kinase; GLUT4, Glucose Transporter Type 4; TPC, total phenolic content; GAE, gallic acid equivalent; HPLC, high performance liquid chromatography; DMEM, dulbecco’s modified eagle medium; FBS, fetal bovine serum; MTT, 3-(4,5 dimethylthiazol-2-yl)-2,5-diphenyltetrazolium bromide; RFU, relative fluorescence units; ANOVA, analysis of variance; SD, standard deviation; VC, ascorbic acid.
